# Overexpression of a Plasma Membrane H^+^-ATPase Gene *OSA1* Stimulates the Uptake of Primary Macronutrients in Rice Roots

**DOI:** 10.3390/ijms232213904

**Published:** 2022-11-11

**Authors:** Ming Ding, Maoxing Zhang, Zihui Wang, Xin Yu, Toshinori Kinoshita, Houqing Zeng, Yiyong Zhu

**Affiliations:** 1 Jiangsu Collaborative Innovation Center for Solid Organic Waste Resource Utilization, College of Resources and Environment Sciences, Nanjing Agricultural University, Nanjing 210095, China; 2 Graduate School of Science, Nagoya University, Nagoya 4660824, Japan; 3 International Research Centre for Environmental Membrane Biology, Department of Horticulture, Foshan University, Foshan 528000, China; 4 Institute of Transformative Bio-Molecules (WPI-ITbM), Nagoya University, Nagoya 4660824, Japan; 5 College of Life and Environmental Sciences, Hangzhou Normal University, Hangzhou 311121, China

**Keywords:** plasma membrane H^+^-ATPase, rice, nitrogen, phosphorus, potassium, transport, noninvasive microtest technology

## Abstract

Plasma membrane (PM) H^+^-ATPase is a master enzyme involved in various plant physiological processes, such as stomatal movements in leaves and nutrient uptake and transport in roots. Overexpression of *Oryza sativa* PM H^+^-ATPase 1 (*OSA1*) has been known to increase NH_4_^+^ uptake in rice roots. Although electrophysiological and pharmacological experiments have shown that the transport of many substances is dependent on the proton motive force provided by PM H^+^-ATPase, the exact role of PM H^+^-ATPase on the uptake of nutrients in plant roots, especially for the primary macronutrients N, P, and K, is still largely unknown. Here, we used *OSA1* overexpression lines (*OSA1*-oxs) and gene-knockout *osa1* mutants to investigate the effect of modulation of PM H^+^-ATPase on the absorption of N, P, and K nutrients through the use of a nutrient-exhaustive method and noninvasive microtest technology (NMT) in rice roots. Our results showed that under different concentrations of P and K, the uptake rates of P and K were enhanced in *OSA1*-oxs; by contrast, the uptake rates of P and K were significantly reduced in roots of *osa1* mutants when compared with wild-type. In addition, the net influx rates of NH_4_^+^ and K^+^, as well as the efflux rate of H^+^, were enhanced in *OSA1*-oxs and suppressed in *osa1* mutants under low concentration conditions. In summary, this study indicated that overexpression of *OSA1* stimulated the uptake rate of N, P, and K and promoted flux rates of cations (i.e., H^+^, NH_4_^+^, and K^+^) in rice roots. These results may provide a novel insight into improving the coordinated utilization of macronutrients in crop plants.

## 1. Introduction 

Plasma membrane (PM) H^+^-ATPase, belonging to the P-type ATPase superfamily, is highly conserved in plant species [1]. In rice, there are ten PM H^+^-ATPase genes (*OSA1–OSA10*). PM H^+^-ATPases generate membrane potential and provide proton motive force for active transport of nutrients, sugars, and other substances. PM H^+^-ATPase activities are regulated at multiple levels, including transcriptional, translational, and post-translational regulations [2]. Environmental stresses, including salinity, alkalinity, nutrient deficiency, and low pH, have been known to activate PM H^+^-ATPases [3,4,5]. The phosphorylation of PM H^+^-ATPase at the penultimate threonine (Thr) residue activates the pump by creating a binding site for 14-3-3 proteins [6,7,8]. Phosphorylation at the penultimate Thr of PM H^+^-ATPases is involved in regulating a variety of physiological processes, such as stomatal opening, hypocotyl elongation, and root growth [8,9,10,11]. While the phosphorylation of PM H^+^-ATPase plays important roles in regulating the physiological functions of plants, the transcriptional regulation of PM H^+^-ATPase genes remains the fundamental basis for controlling PM H^+^-ATPase activity, which is essential for the transmembrane transport of mineral nutrients.

Plants require nitrogen (N), phosphorus (P), and potassium (K) to grow and maintain a variety of metabolic processes [12,13,14]. PM H^+^-ATPase is required for low P adaptation in plants such as soybeans [15]. As a result of P deficiency in white lupin, PM H^+^-ATPase activity is induced in cluster roots, which is associated with citrate release and rhizosphere acidification [16]. Overexpression of a tomato PM H^+^-ATPase gene, *SlHA8*, promotes N and P uptakes in tomato roots [17]. Furthermore, it has been demonstrated that K^+^ transport is chemically linked to ATP hydrolysis [18]. PM H^+^-ATPase provides a driving force for cellular K^+^ uptake mediated by K^+^ channels or H^+^/K^+^ symporters. PM H^+^-ATPase energizes multiple ion channels and various H^+^-coupled transporters, such as HAK1 (a high affinity K^+^ transporter), PHTs (H^+^-coupled phosphate transporters), and NRTs (H^+^/NO_3_^−^ symporters) [19], by generating H^+^ gradient across the PM and membrane potential to drive the transport of these nutrient ions [20]. Several isoforms of PM H^+^-ATPase in rice have been demonstrated to mediate N, P, and K uptakes. For example, NO_3_^−^ was found to induce expression of *OSA2*, *OSA5*, *OSA7*, and *OSA8* in rice roots [21]. Rice *osa8* knockout mutants showed reduced P concentration in the shoots [3]. In some cases, overexpression of *OsHAK5*, a potassium transporter gene, improved PM H^+^-ATPase activity [22]. In our previous studies, we found that overexpression of the *OSA1* gene could improve the NH_4_^+^ uptake rate, increase plant growth, and enhance the total nutrient amount per plant, such as P and K [23]. However, we did not determine the P and K uptake rates in previous studies, and it is still ambiguous whether *OSA1* gene overexpression could stimulate P and K uptakes. In this study, we first estimated the N, P, and K uptake rates of *OSA1* overexpression plants and knockout mutant plants by cultivating them with hydroponic culture under different concentrations of P and K by using nutrient-exhaustion methods. We also used noninvasive microtest technology (NMT) methods to analyze the flux rates of NH_4_^+^, K^+^, and H^+^ with *OSA1* overexpression plants and knockout mutant plants under the high-affinity transport conditions. We found that the overexpression of *OSA1* significantly improved P and K uptake rates in roots. This study could help to demonstrate the crucial role of PM H^+^-ATPases in coordinating N, P, and K absorptions.

## 2. Results

### 2.1. OSA1-Overexpression Promotes Rice Growth under Different Concentrations of P and K

**Figure 1:**
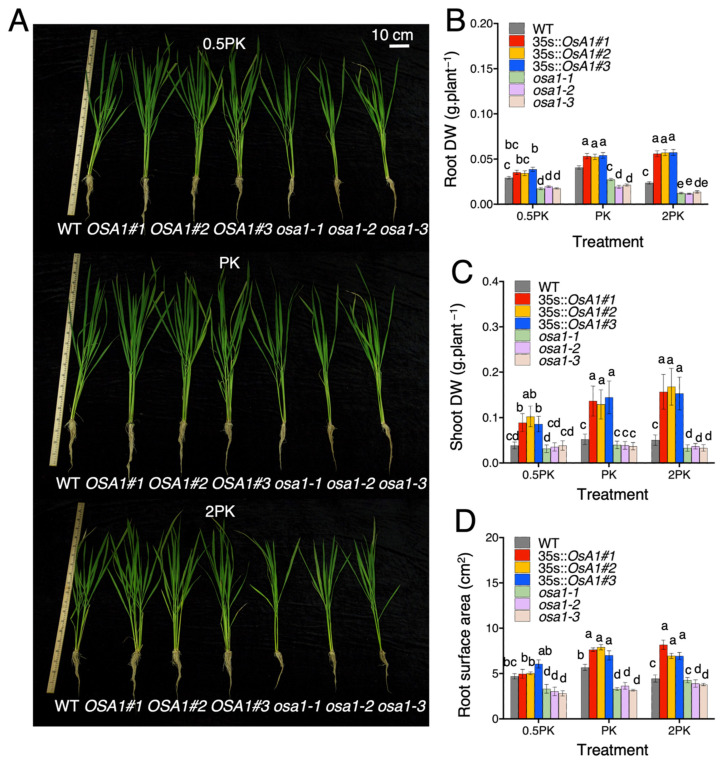
Phenotype of WT, *OSA1*-oxs, and *osa1* mutants. Rice plants were grown hydroponically in a greenhouse for 4 weeks under a 16 h fluorescent light (400 μmol m^−2^ s^−1^)/8 h dark cycle at 24 °C, in 55–70% relative humidity. (**A**) Seedlings were incubated in IRRI nutrient solution containing 2 mM NH_4_^+^ as N source, and half (0.5PK), normal (PK), and twofold (PK) concentrations of P and K. Scale bar = 10 cm. Root dry weight (**B**), shoot dry weight (**C**), and root surface area (**D**) were determined using 5-week-old plants grown in 1/2 IRRI nutrient solution for 1 week, then transferred to full IRRI nutrient solution containing different concentrations of P and K (0.5PK, PK, 2PK) for 4 weeks. Values are the mean ± SD (*n* = 9). Different letters indicate significant differences at *p* < 0.05 according to two-way ANOVA with Tukey’s multiple-comparisons test.

Hydroponically grown *OSA1* overexpression lines (*OSA1*-oxs), *osa1* mutants, and WT rice plants were investigated for phenotypic differences under different concentrations of P and K (normal concentrations of P and K (PK treatment), 0.5-fold P and 0.5-fold K (0.5PK treatment), and 2-fold P and 2-fold K (2PK treatment))*. OSA1*-oxs exhibited improved root and shoot growth when compared with WT (Figure 1A). Under PK treatment, root biomass of *OSA1*-oxs was 30% higher than that of WT. However, when supplied with 2PK, the root biomass reached to 130% higher than that of WT (Figure 1B). In addition, *osa1* mutants showed reduced root biomass by approximately 37–44% under 0.5PK treatment when compared with WT (Figure 1B). It is interesting that the root dry weight of WT under 2PK treatment decreased by 41% compared with that of control (PK treatment), whereas *OSA1*-oxs did not exhibit any growth defects (Figure 1B). These results suggest that high concentrations of P and K inhibit root growth, but higher activities of PM H^+^-ATPases could alleviate this inhibition. The shoot biomass also significantly increased in *OSA1*-oxs under all treatments but decreased in *osa1* mutants under 2PK treatment when compared with WT (Figure 1C). Furthermore, we determined the root surface area of *OSA1*-oxs and *osa1* mutants using root scanner; the root surface area is positively correlated with root dry weight. Under PK and 2PK treatments, the root surface area of *OSA1*-oxs was significantly higher than that of WT, and the root surface area of *osa1* mutants was reduced under 0.5PK and PK treatments (Figure 1D). These results demonstrate that overexpression of *OSA1* can improve plant growth under moderate, low, and high concentrations of P and K. 

### 2.2. PM H^+^-ATPase Activity in Rice Roots of OSA1-Modified Plants under Different Concentrations of P and K

**Figure 2:**
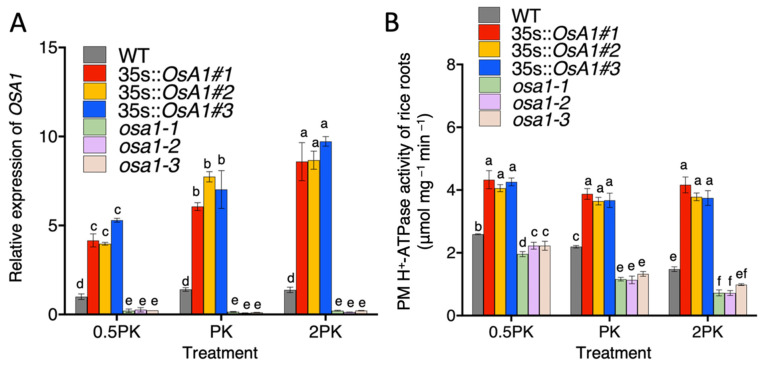
*OSA1* expression level and PM H^+^-ATPase activity in WT, *OSA1*-oxs, and *osa1* mutants under different concentrations of P and K (0.5PK, PK, and 2PK). (**A**) Relative expression of *OSA1* in WT and *OSA1* transgenic plants roots. Values indicate means ± SD (*n* = 3). (**B**) Hydrolytic activity of PM H^+^-ATPase in roots of *OSA1*-oxs and *osa1* mutants. Different letters indicate significant differences at *p* < 0.05 according to two-way ANOVA with Tukey’s multiple-comparisons test.

We next performed qRT-PCR assay to determine *OSA1* gene expression levels in WT, *OSA1*-oxs, and *osa1* mutants under different concentrations of P and K. Results showed that the relative expression levels of *OSA1* were significantly enhanced in the roots of *OSA1*-oxs but were decreased in *osa1* mutants as compared with WT under different concentrations of P and K (Figure 2A). Further investigation of rice root PM H^+^-ATPase activity was conducted under different concentrations of P and K. Results showed that PM H^+^-ATPase activity of WT increased by 18% under the 0.5PK treatment and was suppressed by approximately 33% under the 2PK treatment compared to that of the PK treatment (Figure 2B), which indicates that high concentrations of P and K inhibit PM H^+^-ATPase activity. In addition, *OSA1*-oxs showed significantly higher PM H^+^-ATPase hydrolysis activity under all treatments when compared with WT. By contrast, *osa1* mutants showed significantly reduced PM H^+^-ATPase activity compared to WT under the 0.5 Pk and PK treatments (Figure 2B). 

### 2.3. Overexpression of OSA1 Stimulates P, K, and N Uptake Rates under Different Concentrations of P and K

**Figure 3:**
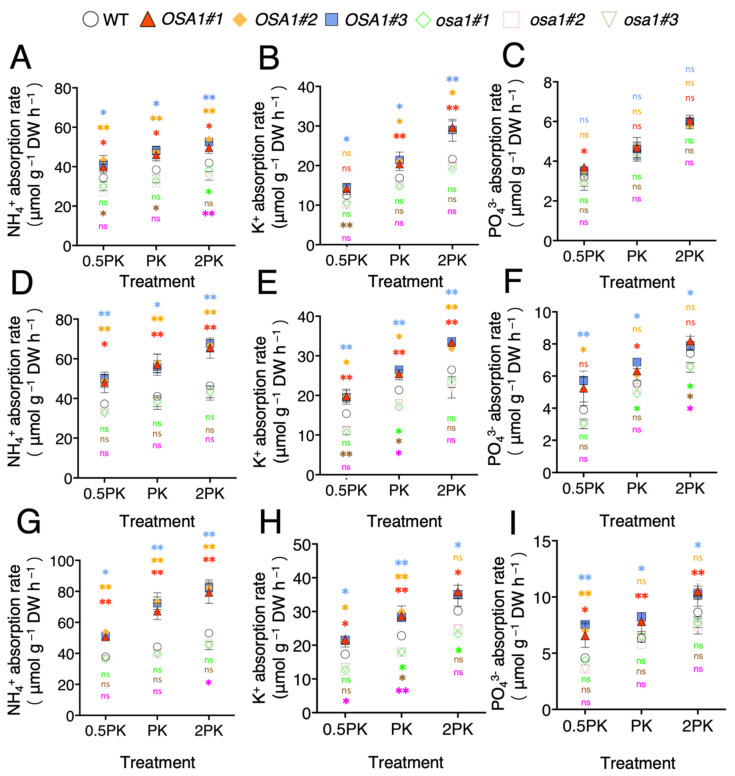
NH_4_^+^, K^+^ and PO_4_^3-^ absorption rates in roots of WT, *OSA1*-oxs, and *osa1* mutants. Rice plants were grown hydroponically in greenhouse for 4 weeks. Seedlings were incubated in IRRI nutrient solutions containing 2 mM NH_4_^+^ as N source, and half (0.5PK), normal (PK), and twofold (2PK) concentrations of P and K for 1 h (**A**–**C**), 2 h (**D**–**F**), and 4 h (**G**–**I**). Values are the mean ± SD (*n* = 3). Differences between *OSA1*-oxs and WT, and between *osa1* mutants and WT were assessed using the two-tailed Student’s *t*-test (ns, no significant difference, * *p* < 0.05; ** *p* < 0.01).

Using the nutrient-exhaustion method, we examined the uptake rates of N, P, and K in rice roots after incubation in a nutrient solution with different concentrations of P and K for 1, 2, and 4 h. As a result of incubation for one hour, the N uptake rate averagely increased by approximately 20.7%, 22.8%, and 24% in *OSA1*-oxs roots, and averagely decreased by approximately 16.1%, 14.7%, and 13.2% in *osa1* mutant roots under the 0.5PK, PK, and 2PK treatments, respectively (Figure 3A). *OSA1*-oxs roots absorbed K with an average rate of about 14.2%, 24.5%, and 34.9% higher than that of WT under the 0.5PK, PK, and 2PK treatments, respectively, but the uptake rate showed no significant difference in *osa1* mutants (Figure 3B). The P uptake rate increased averagely by 11.5% in *OSA1*-oxs under the 0.5PK treatment and there was no significant difference between the *osa1* mutants and WT under all treatments (Figure 3C). After incubation for 2 h, the N uptake rate of *OSA1*-oxs roots averagely increased by approximately 31.3%, 38.4%, and 43.7% under the 0.5PK, PK, and 2PK treatments, respectively, and it exhibited no significant difference in *osa1* mutant roots (Figure 3D). The average K absorption rate of *OSA1*-oxs roots was 27.4%, 20.5%, and 24.6% higher under 0.5PK, PK, and 2PK treatments, respectively, whereas there was no difference between *osa1* mutants and WT (Figure 3E). The average P uptake rate increased by 38%, 15.8, and 8.4% in *OSA1*-oxs under 0.5PK, PK, and 2PK treatments, respectively, and no significant difference was found in *osa1* mutants (Figure 3F). After incubation for 4 h, N uptake rate by *OSA1*-oxs roots averagely increased by approximately 36.7%, 60.4%, and 53.5% under 0.5PK, PK, and 2PK treatments, respectively, and no significant difference was found in *osa1* mutant roots (Figure 3G). In addition, the average K absorption rate of *OSA1*-oxs roots was 23.3%, 27%, and 17.2% higher than WT, and was reduced by 26.6%, 21.3%, and 19.5% in *osa1* mutant roots under 0.5PK, PK, and 2PK treatments, respectively (Figure 3H). As shown in Figure 3I, the average P uptake rate increased by 54%, 25.2%, and 19.9% in *OSA1*-oxs under 0.5PK, PK, and 2PK treatment, respectively. These results indicate that overexpression of *OSA1* could improve macronutrient uptake rates under moderate, low, and high P and K concentrations. 

After 1, 2, and 4 h of incubation at different concentrations of P and K, we determined the total amount of N, P, and K in the whole plants. Under 0.5PK, PK, and 2PK treatments, the NH_4_^+^ amount averagely increased by 114%, 140%, and 165%, respectively, in *OSA1*-oxs and decreased by 42.4%, 28.5%, and 29.4%, respectively, in *osa1* mutants compared to WT (Figure S1A). P content averagely increased by 78%, 103%, and 124% in *OSA1*-oxs, and averagely decreased by 33.8%, 34.7%, and 31.7% in *osa1* mutants under 0.5PK, PK, and 2PK treatments, respectively (Figure S1B). In addition, the K content in *OSA1*-oxs averagely increased by 119%, 115%, and 112%, and averagely decreased by 24.8%, 40.3%, and 41% in *osa1* mutants when compared with WT under 0.5PK, PK, and 2PK treatments, respectively (Figure S1C). 

### 2.4. Overexpression of OSA1 Increases the Expression of Nutrient Transporter Genes in Roots under Different Concentrations of P and K 

**Figure 4:**
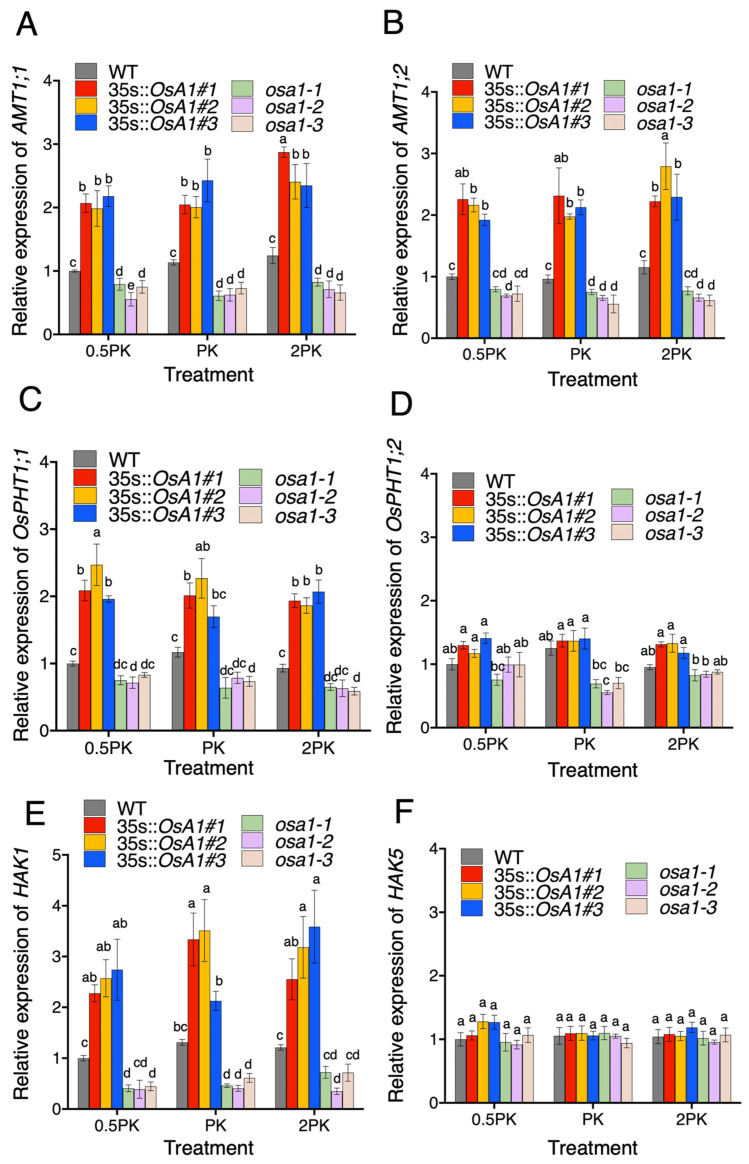
Relative expression level of N, P, and K transporter genes in roots of WT, *OSA1*-oxs, and *osa1* mutants. Four-week-old rice plants were cultured in IRRI nutrient solution containing 2.0 mM NH_4_^+^ and half (0.5PK), normal (PK), and twofold (2PK) concentrations of P and K for 4 h. (**A**,**B**) Relative expression levels of *AMT1;1* and *AMT1;2*. (**C**,**D**) Relative expression levels of *OsPHT1;1* and *OsPTH1;2*. (**E**,**F**) Relative expression levels of *HAK1* and *HAK5*. Values are presented as the means ± SD (*n* = 3). Different letters indicate significant differences at *p* < 0.05 according to two-way ANOVA with Tukey’s multiple-comparisons test.

**Figure 5:**
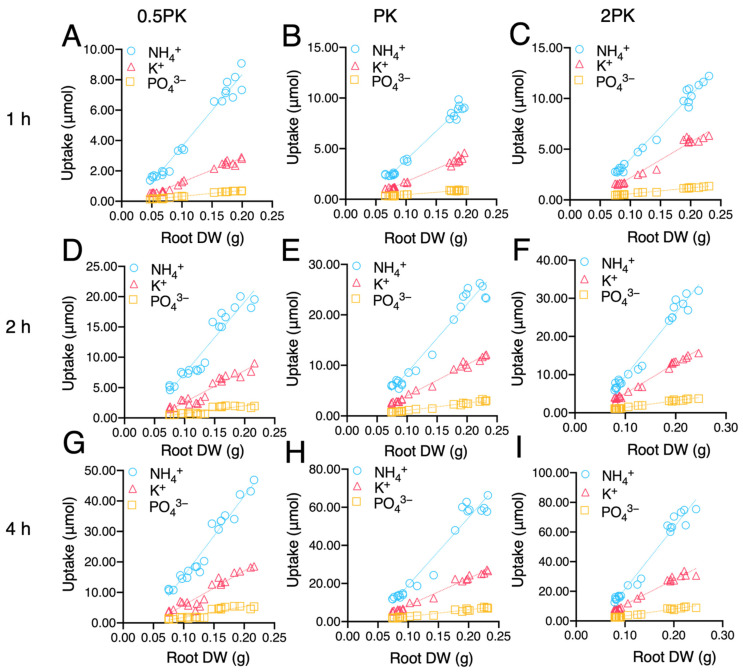
Correlation between N, P, and K uptake contents and root dry weight (DW). Rice seedlings including WT, *OSA1*-oxs, and *osa1* mutants were incubated under 0.5PK (**A**,**D**,**G**), PK (**B**,**E**,**H**), and 2PK (**C**,**F**,**I**) IRRI nutrient solutions, with 2.0 mM NH_4_^+^ being the N source for 1 h (**A**–**C**), 2 h (**D**–**F**), and 4 h (**G**–**I**). The difference of N, P, and K contents between blank solution and incubation solution was used to calculate for the nutrient uptake content by roots.

Next, we determined the relative expression levels of the corresponding genes related to N, P, and K uptakes under different concentrations of P and K in *OSA1* overexpression and knockout mutant plants by qRT-PCR assay. *OsAMT1;1* and *OsAMT1;2* are two critical NH_4_^+^ transporter genes that are highly induced by N deficiency [24]. Here, we found that the relative expression level of *OsAMT1;1* was about 2–3 times higher in *OSA1*-oxs roots and decreased significantly in *osa1* roots when compared with that of WT under all treatments of different concentrations of P and K (Figure 4A). In addition, the relative expression level of *OsAMT1;2* in *OSA1*-oxs roots was also significantly higher than that of WT under all these conditions and decreased significantly in *osa1* mutants under the PK condition (Figure 4B). *OsPHT1;1* and *OsPHT1;2*, belonging to OsPHT gene family, are involved in phosphate uptake in rice roots [25]. Here, we found that *OsPHT1;1* expression was significantly enhanced in *OSA1*-oxs roots but showed no significant difference in *osa1* mutants when compared with WT (Figure 4C). However, the expression of *OsPHT1;2* showed no significant difference among *OSA1*-oxs, *osa1* mutants, and WT (Figure 4D). Then, we detected expression levels of K transporter genes, *HAK1* and *HAK5* [26]. *OSA1*-oxs plants exhibited at least a two times higher expression level of *HAK1* in the roots under all these treatments, and *osa1* mutants showed a reduced expression level of *HAK1* under PK treatment (Figure 4E), but no significant difference was found for *HAK5* (Figure 5F). All these results suggest that the overexpression of *OSA1* could activate the expression of N, P, and K transporter genes under moderate, low, and high P and K concentrations.

### 2.5. Correlation between N/P/K Uptakes and Root Morphology

A correlation analysis was conducted between N, P, and K uptakes and root morphology. These nutrient uptakes were all positively correlated with root dry weight and root surface area (Figure 5 and Figure S2). Interestingly, the increment trend of nutrient uptake with root dry weight or root surface area was in the order of N > K > P under all treatments (Figure 5 and Figure S2). The R^2^ values and regression equations for their correlations were shown in Tables S1 and S2. The correlation coefficients were all significant at *p* value < 0.001. According to these results, improved NH_4_^+^, PO_4_^3-^, and K^+^ uptake could not only attribute by the enhanced PM H^+^-ATPase activity, but also by the increased root growth conferred by *OSA1* overexpression.

### 2.6. OSA1 Overexpression Promotes Cation Fluxes in Rice Roots under High-Affinity Transport Concentrations

**Figure 6:**
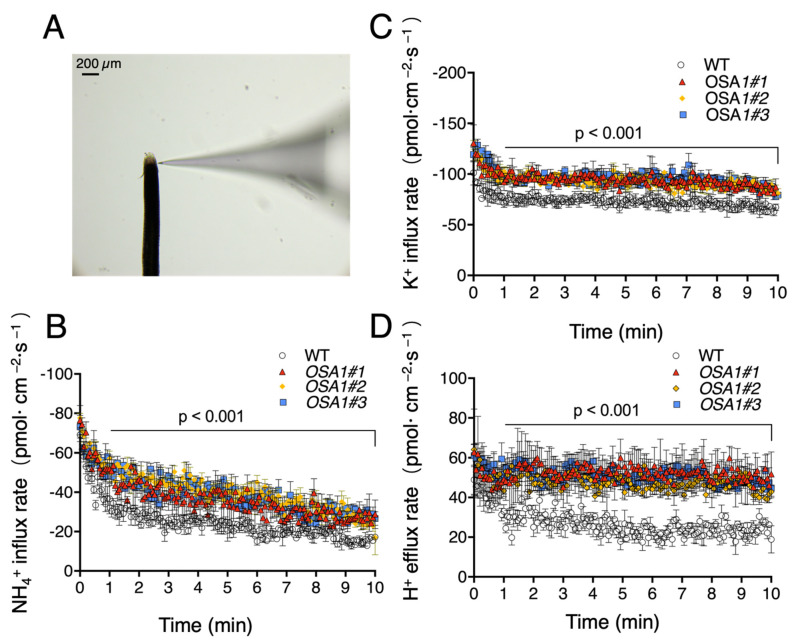
Quantification of NH_4_^+^ influx, K^+^ influx, and H^+^ efflux rates of WT and *OSA1*-oxs roots. The 5-day-old seedlings of WT and *OSA1*-oxs were treated with hydroponic solution lacking NH_4_^+^ or K^+^ for 24 h. Intact roots were equilibrated in the measuring solution (0.1 mM NH_4_^+^ and 0.1 mM K^+^ are in the test solution) for 10 min. (**A**) The nutrient flux rate was measured along the root tip, around 150 μm from the root cap. Black-colored object is a rice root and the right one is a microelectrode probe used to determine the change of ion flux in the root surface. The NH_4_^+^ influx rate (**B**), K^+^ influx rate (**C**), and H^+^ influx rate (**D**) were calculated. Values are mean ± SD (*n* = 3). Significant difference was evaluated using the two-tailed Student’s *t* test.

**Figure 7:**
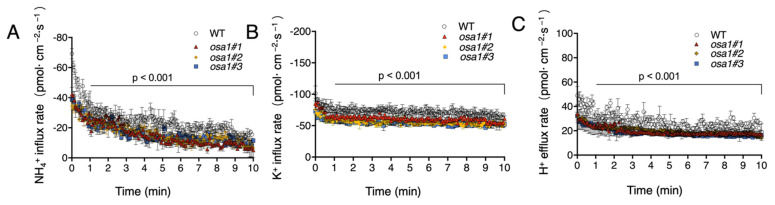
Quantification of NH_4_^+^ influx, K^+^ influx, and H^+^ efflux rates of WT and *osa1* mutant roots. The 5-day-old seedlings of WT and *osa1* mutants were treated with a hydroponic solution lacking NH_4_^+^ for 24 h. Intact roots were equilibrated in the measuring solution for 10 min. The NH_4_^+^ influx rate (**A**), K^+^ influx rate (**B**), and H^+^ efflux rate (**C**) were tested. Values are means ± SD (*n* = 3). Significant difference was evaluated using the two-tailed Student’s *t*-test.

To measure the net flux rates of NH_4_^+^, K^+^, and H^+^ in roots of WT and *OSA1* transgenic plants under high-affinity transport concentrations of NH_4_^+^ and K^+^ (0.1 mM NH_4_^+^ and 0.1 mM K^+^), we used a noninvasive microtest system (NMT) to perform the microelectrode ion flux measurement (MIFE) with intact roots of 5-day-old seedlings. The nutrient flux rate was measured along the root tip, around 150 μm from the root cap (Figure 6A). As a result, the NH_4_^+^ influx rate, K^+^ influx rate, and H^+^ efflux rate increased in three *OSA1*-oxs by 64.2%, 28.3%, and 96.1%, respectively (Figure 6B–D), and decreased significantly in the roots of three osa1 mutants by approximately 35.7%, 27.3%, and 21%, respectively, when compared with that of WT (Figure 7A–C). These results suggest that *OSA1* plays a positive role in controlling NH_4_^+^ and K^+^ uptake rates, as well as the H^+^ excretion rate in rice roots under a high-affinity transport system.

## 3. Discussion

Numerous studies have been conducted on the effects of N, P, and K on crop growth and development [27,28]. Our previous study demonstrated that the overexpression of *OSA1* significantly enhanced NH_4_^+^ uptake and improved grain yield in rice [21]. The present result showed that the overexpression of *OSA1* in rice significantly increased the PM H^+^-ATPase activity (Figure 2B), and activated the expression of several nutrient transporter genes, such as *OsAMT1;1*, *OsAMT1;2*, *OsPHT1;1*, and *OsHAK1* (Figure 4), and improved the uptake rates of macronutrients such as N, P, and K by roots under different concentrations of P and K (Figure 3). 

Notably, the 0.5PK treatment activated PM H^+^-ATPase in WT roots compared with that of normal conditions (PK treatment) (Figure 2B), which is consistent with previous findings that low K^+^ or low P concentrations in apoplast could significantly improve PM H^+^-ATPase activity [3,29]. In addition, we found higher concentrations of P and K improved NH_4_^+^ uptake rates in WT, and this effect was even enhanced in *OSA1*-oxs plants (Figure 3A,D,G). Previous studies showed that a high K concentration reduced the NH_4_^+^ uptake rate in rice due to the competitive absorption between K^+^ and NH_4_^+^ [30]. It is possible that the increased uptake of phosphate could help to maintain the charge balance in the cytoplast and further facilitate the uptake of K^+^. Theoretically, absorption of N, P, and K will increase H^+^ concentrations in root cytoplasm, which need to be pumped out of cells by PM H^+^-ATPase to maintain the membrane potential and cytosolic pH. Interestingly, overexpression of *OSA1* activated the expression of several transporter genes related to N, P, and K uptakes in rice under low-affinity P and K conditions (Figure 4). However, the underlying molecular mechanism of the linkage between PM H^+^-ATPase activity and the expression of nutrient transporter genes deserves to be investigated in the future. 

PM H^+^-ATPase has been known to be involved in auxin-induced root growth in the model plant Arabidopsis [9,10,31,32]. In rice, it has been demonstrated that overexpression of *OSA1* enhanced root biomass under different concentrations of NH_4_^+^ [21]. In this study, we found that biomass also increased significantly under different concentrations of P and K in *OSA1*-oxs and decreased in *osa1* mutants (Figure 1). Interestingly, we found that 2PK treatment significantly inhibited root growth in WT and *osa1* mutants, but the growth inhibition was alleviated in *OSA1*-oxs (Figure 1). Increases in the uptakes of P and K may lead to cytosolic H^+^ accumulation and even H^+^ toxicity in the cytoplast of rice roots, which could negatively affect root growth [33,34]. However, improved PM H^+^-ATPase activity by overexpression of *OSA1* could alleviate H^+^ accumulation in root cells and then contribute to the tolerance to high concentrations of P and K. In addition, N, P, and K contents in *OSA1*-oxs showed linear relationships with root biomass and root surface area (Figure 5 and Figure S2), which is consistent with previous studies which found that nutrient uptake amounts are correlated with root biomass [35,36]. However, the underlying mechanism of how PM H^+^-ATPase activity is linked to the root growth of rice under different nutrient concentrations needs to be investigated in the future, which may possibly be associated with auxin signaling.

In the high-affinity transport system, it has been well documented that PM H^+^-ATPase is involved in nutrient uptakes by supplying proton motive force to facilitate ion transports across the PM [37]. However, the detailed information is unclear about the effect of PM H^+^-ATPase overexpression on nutrient transportation in rice roots. Here, we performed MIFE experiment with NMT system to measure the net ion fluxes around the root tips of *OSA1*-modified plants and WT. In the root tip region, comprising the meristem and elongation zone (0–600 µm from the tip), a strong ion flux can be detected [38]. In this study, we detected one at the position of 150 µm far from the root tip, which belongs to the elongation region close to the meristem region (Figure 6A). The results showed that the net NH_4_^+^ and K^+^ influx rates were significantly increased in *OSA1*-oxs and were significantly reduced in *osa1* mutants under the high-affinity transport system (0.1 mM NH_4_^+^ and 0.1 mM K^+^) when compared with that of WT (Figure 6 and Figure 7). NMT uses specialized flux sensors that are derived from microelectrodes to measure the dynamic ion/molecule activity (i.e., diffusion flux). Diffusion flux is the amount of substance per unit area per unit time around an intact sample [39]. Therefore, the overexpression of *OSA1* improved nutrient uptake rate directly, possibly by generating more polarized membrane potential, which is independent of the root biomass. However, to our knowledge, NMT methods are not permitted for determining phosphate influx rates in rice roots, which is expected to be explored in the future.

In conclusion, we demonstrated that the overexpression of a rice PM H^+^-ATPase gene, *OSA1*, promoted root growth and stimulated N, P, and K uptake rates by roots under different concentrations of P and K in rice. Actually, this report firstly provided evidence that the engineering of a PM H^+^-ATPase gene in rice could improve plant growth under moderate, low, and high P and K concentrations. This study could also provide a novel insight into the cultivating crop plants growing in agricultural soil, either with inadequate abundance of nutrients or with excessive abundance of nutrients. Further studies are required to explore the underlying mechanism by which *OSA1* overexpression enhances root growth and nutrient uptake under moderate, low, and high concentrations of P and K. 

## 4. Materials and Methods 

### 4.1. Plant Cultivation

All the rice seeds used in this study were kept in our own lab [21]. Seeds of WT (Oryza sativa L. ssp. japonica cv. Nipponbare); overexpression lines of *OSA1#1*, *OSA1#2*, and *OSA1#3*; and mutant lines *osa1-1* (TOS17 line ND3017), *osa1-2* (TOS17 line ND3025), and *osa1-3* (TOS17 line ND3033) were surface-sterilized in 10% H_2_O_2_ for 30 min and then soaked in water before germination. Rice seedlings were grown in a greenhouse under a light intensity of 400 μmol m^−2^ s^−1^, a relative humidity of approximately 60–80%, and a 14 h light (30 °C)/10 h dark (22 °C) photoperiod. Pots with PO_4_^3−^ and K^+^ treatments were filled with a minor modified IRRI nutrient solution as follows: 0.5PK treatment contains 0.25 mM K_2_SO_4_, and 0.15 mM NaH_2_PO_4_; 2PK treatment contains 1 mM K_2_SO_4_, and 0.6 mM NaH_2_PO_4_, and PK treatment (control) contains 0.5 mM K_2_SO_4_ and 0.3 mM NaH_2_PO_4_. The full chemical composition of the IRRI nutrient solution (pH 5.5) is listed below: 1 mM (NH_4_)_2_SO_4_, 0.5 mM K_2_SO_4_, 0.3 mM NaH_2_PO_4_, 1 mM CaCl_2_, 1 mM MgSO_4_, 9 μM MnCl_2_, 0.39 μM Na_2_MoO_4_, 20 μM H_3_BO_4_, 0.77 μM ZnSO_4_, 0.32 μM CuSO_4_, and 20 μM EDTA-Fe [21]. Solutions were changed every 3 days. Rice seedlings were grown in 1/2 IRRI nutrient solution for 1 week, then transferred to full IRRI nutrient solution containing different concentrations of P and K (0.5PK, PK, 2PK) for 4 weeks.

### 4.2. Nutrient Uptake Rate Determination

Three plants exhibiting similar growth in each treatment were used for the exhaustion test according to [40,41] with some modifications. The rice seedlings were placed in a N-, P-, and K-free nutrient solution for 24 h, then plants were transferred into 250 mL IRRI nutrient solutions with different concentrations of P and K to analyze nutrient uptake rates. All experiments were performed in a growth chamber at 28 °C with a light intensity of 400 μmol m^−2^ s^−1^ and relative humidity of 60%. The culture medium was sampled at 1 h, 2 h, 4 h. After that, one mL of exhaustion fluid was used for the determination of the N, P, and K concentration and was replenished with 1 mL of deionized water at the same time to ensure that the volume of depleted fluid was constant. Control solution was used as negative control, which contained the same nutrient solution with the treatment but without root incubation. At the end of the exhaustion test, the fresh root weight was calculated after the moisture was removed with filter paper. K concentration in the exhaustion fluid and control solution was measured by flame emission photometry. P concentration was determined using the molybdate yellow method. The concentration of NH_4_^+^ was determined by the continuous-flow analytical system AA3 (SEAL, Gernany) (The detection limit is 0.0165 mg·L^−1^). 

### 4.3. Measurements of Net Proton, K^+^, and NH_4_^+^ Fluxes Rate in Roots

A BIO-IM Series NMT Physiolyzer^®^ system (YoungerUSA, MA, USA) was applied to determine the net H^+^, K^+^, and NH_4_^+^ fluxes in the root tip based on a previous study [42]. Five-day-old rice roots were subsequently placed in 50 mL of growth solution with 2 mM NH_4_^+^ for 12 h. After washing with deionized water, the roots were placed in Petri dishes containing 20 mL detection buffer containing 0.2 mM CaCl_2_, 0.1 mM KCl, 0.1 mM MgCl_2_, 0.5 mM NaCl, 0.3 mM MES (2-morpholinoethanesulfonic acid sodium salt), and 0.2 mM Na_2_SO_4_ (pH 6.0) for the determination of the H^+^ and K^+^ fluxes. For the NH_4_^+^ flux assay, the detection buffer containing 0.1 mM NH_4_NO_3_, 0.1 mM KCl, 0.1 mM CaCl_2_, and 0.3 mM MES (pH 6.0) was used. The fluxes of rice plants were measured along the root tip around 150 μm from the root cap. The microelectrodes in the NMT system were positioned 0 ± 2 μm away from the samples. At least three individual plants were analyzed for each sample.

### 4.4. Quantitative Reverse-Transcription PCR

qRT-PCR was performed using the Step One Real-Time PCR system (Applied Biosystems, Foster City, CA, USA), as described previously [43]. Briefly, total RNA was extracted from the roots of 4-week-old WT and transgenic plants using the RNeasy Plant Mini Kit (Qiagen). First-strand cDNAs were synthesized from total RNA using the PrimeScript II First Strand cDNA Synthesis Kit using oligo (dT) primers (TakaRa). Relative quantification was performed using an SYBR Premix Ex Taq II (Perfect Real Time) Kit (TaKaRa Biotechnology, Dalian, China) on a Step One Plus Real-Time PCR System (Applied Biosystems, Bio-Rad, CA, USA). For amplification of *OSA1* (Os03g0689300), *OsAMT1;1* (Os04g0509600), *OsAMT1;2* (Os02g0620600), *OsPHT1;1* (Os03g0150600), *OsPHT1;2* (Os03g0150800), *OsHAK1* (Os04g0401700), and *OsHAK5* (Os01g0930400), the gene-specific primers were used (Table S3). Relative gene expression levels were normalized to that of the internal control gene, *OsActin* (Os03g0718100), using the comparative cycle threshold (ΔΔCt) method [44]. All analyses were repeated at least three times.

### 4.5. Measurement of PM H^+^-ATPase Activity 

The detection of ATP hydrolytic activity of PM H^+^-ATPase was completed mainly according to [16], with a few modifications. Root tissues of 4-week-old WT and transgenic plants treated with different concentrations of P and K (0.5 PK, PK, and 2PK) were ground in ice-cold homogenization buffer to isolate the PM in a two-phase partitioning method. The protein concentration of the membrane vesicle was determined by using Bio-Rad protein assay dye reagent concentrate (BIO-RAD, Hercules, CA, USA) according to the manufacturer’s instruction. Na_3_VO_4_ (0.1 mM) was used as an inhibitor of H^+^-ATPase in 0.5 mL reaction solution, which contained 30 mM BTP/MES, 5 mM MgSO_4_, 50 mM KCl, 50 mM KNO_3_, 1 mM Na_2_MoO_4_, 1 mM NaN_3_, 0.02% (*w*/*v*) Brij 58, and 5 mM disodium-ATP (substrate for PM H^+^-ATPase). Firstly, 30 μL of a membrane vesicle suspension containing 1–2 μg total protein was added to the reaction solution for 30 min at 30 °C; the inorganic phosphate was liberated after the hydrolysis of ATP. The reaction was stopped by adding 1 mL reagent (2% (*v*/*v*) concentrated H_2_SO_4_, 5% (*w*/*v*) sodium dodecyl sulfate, and 0.7% (*w*/*v*) (NH_4_)_2_MoO_4_), followed by 50 μL 10% (*w*/*v*) ascorbic acid. After 10 min, 1.45 mL arsenide-citrate reagent (2% (*w*/*v*) sodium citrate, 2% (*w*/*v*) sodium arsenide, and 2% (*w*/*v*) glacial acetic acid) was added. The color will be stable after 30 min and measured spectrophotometrically at 720 nm. In each test, H^+^-ATPase activity was calculated as the amount of phosphate liberated within 30 min mg^−1^ membrane protein in excess of the boiled-membrane protein control.

### 4.6. Statistical Analysis

Data were analyzed for statistics and significance by two-tailed student’s *t* test and two-way ANOVA test with a significant difference at *p* < 0.05 using GraphPad Prism 9 software (GraphPad Software, San Diego, CA, USA). 

## Data Availability

Not applicable.

## Supplementary Materials

The following supporting information can be downloaded at: https://www.mdpi.com/article/10.3390/ijms232213904/s1.

## References

[1] Arango M., Gévaudant F., Oufattole M., Boutry M. (2003). The plasma membrane proton pump ATPase: The significance of gene subfamilies. Planta.

[2] Heven S., Li X.H., Palmgren M.G. (1999). Energization of Plant Cell Membranes by H^+^-Pumping ATPases: Regulation and Biosynthesis. Plant Cell.

[3] Chang C.R., Hu Y.B., Sun S.B., Zhu Y.Y., Ma G.J., Xu G.H. (2009). Proton Pump *OsA8* Is Linked to Phosphorus Uptake and Translocation in Rice. J. Exp. Bot..

[4] Zhu Y.Y., Di T.J., Xu G.H., Chen X., Zeng H.Q., Yan F., Shen Q.R. (2009). Adaptation of Plasma Membrane H^+^-ATPase of Rice Roots to Low PH as Related to Ammonium Nutrition. Plant Cell Environ..

[5] Cao Y., Zhang M., Liang X. (2020). Natural variation of an EF-hand Ca^2+^-binding-protein coding gene confers saline-alkaline tolerance in maize. Nat. Commun..

[6] Olsson A., Svennelid F., Sommarin M. (1998). A phosphothreonine residue at the c-terminal end of the plasma membrane H^+^-ATPase is protected by fusicoccin-induced 14–3–3 binding1. Plant Physiol..

[7] Fuglsang A.T., Visconti S., Drumm K., Jahn T., Stensballe A., Mattei B., Jensen O.N., Aducci P., Palmgren M.G. (1999). Binding of 14–3–3 protein to the plasma membrane H(+)-ATPase AHA2 involves the three C-terminal residues Tyr(946)-Thr-Val and requires phosphorylation of Thr(947). J. Biol. Chem..

[8] Kinoshita T., Shimazaki K.I. (1999). Blue Light Activates the Plasma Membrane H^+^-ATPase by Phosphorylation of the C-Terminus in Stomatal Guard Cells. EMBO J..

[9] Takahashi K., Hayashi K., Kinoshita T. (2012). Auxin activates the plasma membrane H+-ATPase by phosphorylation during hypocotyl elongation in Arabidopsis. Plant Physiol..

[10] Spartz A.K., Ren H., Park M.E., Grandt K.N., Lee S.H., Murphy A.S., Sussman M.R., Overvoorde P.J., Gray W.M. (2014). SAUR Inhibition of *PP2C-D* Phosphatases Activates Plasma Membrane H^+^-ATPases to Promote Cell Expansion in Arabidopsis. Plant Cell..

[11] Inoue S., Takahashi K., Okumura-Noda H., Kinoshita T. (2016). Auxin influx carrier AUX1 confers acid resistance for Arabidopsis root elongation through the regulation of plasma membrane H+-ATPase. Plant Cell Physiol..

[12] Raghothama K.G. (1999). Phosphate acquisition. Annu. Rev. Plant Physiol. Mol. Biol..

[13] Marschner P. (2012). Marschner’s Mineral Nutrition of Higher Plants.

[14] Li J., Long Y., Qi G., Li J., Xu Z., Wu W., Wang Y. (2014). The Os-AKT1 channel is critical for K^+^ uptake in rice rootsandis modulated by the rice CBL1-CIPK23 complex. Plant Cell.

[15] Shen H., Chen J., Wang Z., Yang C., Sasaki T., Yamamoto Y., Matsumoto H., Yan X. (2006). Root plasma membrane H^+^-ATPase is involved in the adaptation of soybean to phosphorus starvation. J. Exp. Bot..

[16] Yan F., Zhu Y., Müller C., Zörb C., Schubert S. (2002). Adaptation of H^+^-pumping and plasma membrane H+-ATPase activity in proteoid roots of white lupin under phosphate deficiency. Plant Physiol..

[17] Liu J.L., Chen J.D., Xie K., Tian Y., Yan A.N., Liu J.J., Huang Y.J., Wang S.S., Zhu Y.Y., Chen A.Q. (2020). A mycorrhiza- specific H^+^-ATPase is essential for arbuscule development and symbiotic phosphate and nitrogen uptake. Plant Cell Environ..

[18] Briskin D.P., Margaret C.G. (1996). Role of the Plasma Membrane H^+^-ATPase in K^+^ Transport. Plant Physiol..

[19] Ding M., Zhang M.X., Zeng H.Q., Hayashi Y.K., Zhu Y.Y., Kinoshita T. (2021). Molecular basis of plasma membrane H^+^-ATPase function and potential application in the agricultural production. Plant Physiol. Bioch..

[20] Palmgren M.G. (2001). Plant Plasma Membrane H ^+^ -ATPases. Annu. Rev. Plant Physiol. Mol. Biol..

[21] Sperandio M.V.L., Santos L.A., Bucher C.A. (2011). Isoforms of plasma membrane H -ATPase in rice root and shoot are differentially induced by starvation and resupply of NO_3_^-^ or NH_4_^+^. Plant Sci..

[22] Yang T., Feng H., Zhang S. (2020). The potassium transporter OsHAK5 alters rice architecture via ATP-dependent transmembrane auxin fluxes. Plant Commn..

[23] Zhang M.X., Wang Y., Chen X., Xu F.Y., Ding M., Ye W.X., Kawai Y.Y., Toda Y.S., Hayashi Y.K., Suzuki T. (2021). Plasma Membrane H^+^-ATPase Overexpression Increases Rice Yield via Simultaneous Enhancement of Nutrient Uptake and Photosynthesis. Nature Commn..

[24] Bao A., Liang Z., Zhao Z., Cai H. (2015). Overexpressing of OsAMT1–3, a High Affinity Ammonium Transporter Gene, Modifies Rice Growth and Carbon-Nitrogen Metabolic Status. Int. J. Mol. Sci..

[25] Anandan A., Parameswaran C., Mahender A. (2021). Trait variations and expression profiling of OsPHT1 gene family at the early growth-stages under phosphorus-limited conditions. Sci. Rep..

[26] Chen G., Hu Q., Luo L., Yang T., Zhang S., Hu Y., Yu L., Xu G. (2015). Rice potassium transporter OsHAK1 is essential for maintaining potassium-mediated growth and functions in salt tolerance over low and high potassium concentration ranges. Plant Cell Environ..

[27] Milla R., Castro-Díez P., Maestro-Martínez M. (2005). Relationships between phenology and the remobilization of nitrogen, phosphorus and potassium in branches of eight Mediterranean evergreens. New Phytol..

[28] Wang Y., Chen Y.F., Wu W.H. (2021). Potassium and phosphorus transport and signaling in plants. J. Integr. Plant Biol..

[29] Rodríguez-Navarro A. (2000). Potassium Transport in Fungi and Plants. Biochim. Biophys. Acta. Biomembr..

[30] Weng L., Zhang M., Wang K., Chen G., Ding M., Yuan W., Zhu Y., Xu W., Xu F. (2020). Potassium alleviates ammonium toxicity in rice by reducing its uptake through activation of plasma membrane H^+^-ATPase to enhance proton extrusion. Plant Physiol. Biochem..

[31] Hoffmann R.D., Olsen L.I., Ezike C.V., Pedersen J.T., Manstretta R., López-Marqués R.L., Palmgren M. (2019). Roles of plasma membrane proton ATPases AHA2 and AHA7 in normal growth of roots and root hairs in Arabidopsis thaliana. Physiol. Plant.

[32] Ren H., Park M.Y., Spartz A.K., Wong J.H., Gray W.M. (2018). A subset of plasma membrane-localized PP2C.D phosphatases negatively regulate SAUR-mediated cell expansion in Arabidopsis. PLoS Genet..

[33] Shukla D., Rinehart C.A., Sahi S.V. (2017). Comprehensive study of excess phosphate response reveals ethylene mediated signaling that negatively regulates plant growth and development. Sci. Rep..

[34] Xu X., Du X., Wang F., Sha J., Chen Q., Tian G., Zhu Z., Ge S., Jiang Y. (2000). Effects of Potassium Levels on Plant Growth, Accumulation and Distribution of Carbon, and Nitrate Metabolism in Apple Dwarf Rootstock Seedlings. Front. Plant Sci..

[35] Hou L., Zhang X., Feng G. (2021). Arbuscular mycorrhizal enhancement of phosphorus uptake and yields of maize under high planting density in the black soil region of China. Sci. Rep..

[36] Newman E.I., Andrews R.E. (2004). Uptake of phosphorus and potassium in relation to root growth and root density. Plant Soil.

[37] Sondergaard T.E., Schulz A., Palmgren M.G. (2004). Energization of Transport Processes in Plants. Roles of the Plasma Membrane H^+^-ATPase. Plant Physiol..

[38] Jia L., Xie Y., Wang Z., Luo L., Zhang C., Pélissier P.M., Parizot B., Qi W., Zhang J., Hu Z. (2020). Rice plants respond to ammonium stress by adopting a helical root growth pattern. Plant J..

[39] Kühtreiber W.M., Jaffe L.F. (1990). Detection of extracellular calcium gradients with a calcium specific vibrating electrode. J. Cell Biol..

[40] Hu W., Di Q., Wang Z. (2019). Grafting alleviates potassium stress and improves growth in tobacco. BMC Plant Biol..

[41] Wang K., Hu Q., Wei Y., Yin H., Sun C., Liu G. (2021). Uptake Kinetics of NH_4_^+^, NO_3_^-^ and H_2_PO_4_^-^ by Typha orientalis, *Acorus calamus* L.; *Lythrum salicaria* L.; *Sagittaria trifolia* L. and *Alisma plantago-aquatica Linn*. Sustainability.

[42] Xu W., Jia L., Shi W., Liang J., Zhou F., Li Q., Zhang J. (2013). Abscisic acid accumulation modulates auxin transport in the root tip to enhance proton secretion for maintaining root growth under moderate water stress. New Phytol..

[43] Wang Y., Noguchi K., Ono N., Inoue S.I., Terashima I., Kinoshita T. (2014). Overexpression of plasma membrane H^+^-ATPase in guard cells promotes light-induced stomatal opening and enhances plant growth. Proc. Natl. Acad Sci. USA.

[44] Livak K.J., Schmittgen T.D. (2001). Analysis of relative gene expression data using real-time quantitative PCR and the 2(-Delta Delta C(T)) Method. Methods.

