# Supplementary material for: Overexpression of a Plasma Membrane H+-ATPase Gene OSA1 Stimulates the Uptake of Primary Macronutrients in Rice Roots
Source: Int J Mol Sci. 2022 Nov 11;23(22):13904. doi: 10.3390/ijms232213904 (PMC9697395; doi:10.3390/ijms232213904)
Supplement: Supplementary file 1 [file ijms-23-13904-s001.zip › ijms-1978222-supplementary.pdf]

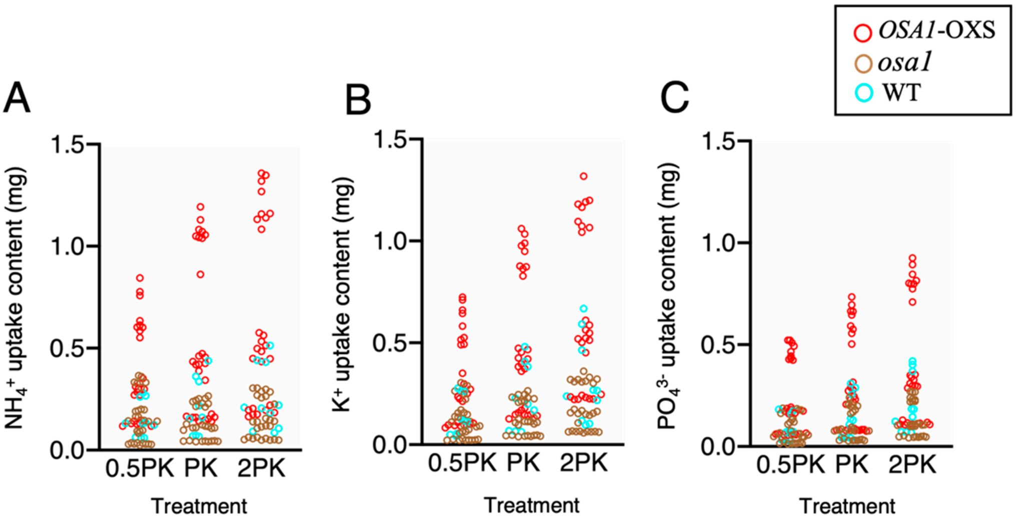

Figure S1. Uptake content of NH_4^+, PO_4^{3-} and K^+ in roots of WT, *OSA1*-oxs, and *osa1* mutants. Rice plants were grown hydroponically in greenhouse for 4 weeks. Seedlings were incubated in IRRI nutrient solutions containing 2 mM NH_4^+ and 0.5PK, PK, or 2PK for 1, 2 and 4 h. (A) NH_4^+ uptake content in rice roots. (B) PO_4^{3-} uptake content in rice roots. (C) K^+ uptake content in rice roots. Small circles indicate data points for each plant collected after incubation for 1 h, 2 h and 4 h.

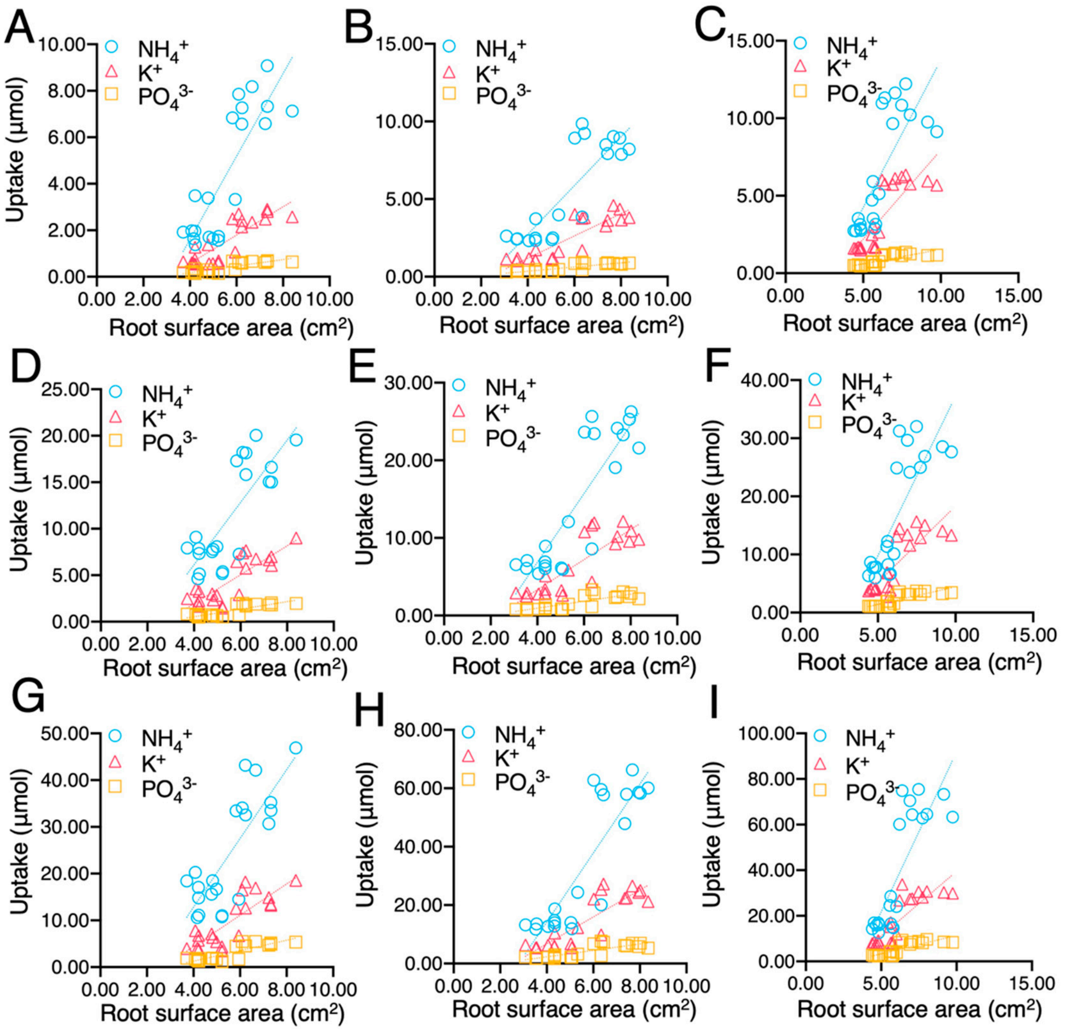

Figure S2. Correlation between NH_4^+, K^+ and PO_4^{3-} uptake content and root surface area. Rice seedlings were incubated under 0.5PK (A, D, and G), PK (B, E, and H) and 2PK (C, F, and I) for 1h (A-C), 2h (D-F) and 4h (G-I). IRRT nutrients solutions were used with 2 mM NH_4^+ as N source. The difference of N, P and K contents between the incubation solution and control solution was used to calculate the nutrient uptake contents of roots.

Table S1. The correlations between nutrient absorptions (NH_4^+, PO_4^{3-} and K^+) and root dry weight under different concentrations of P and K (0.5PK, PK, and 2PK).

Time	Treatment	Matched curve			R^2		
		NH_4^+	PO_4^{3-}	K^+	NH_4^+	PO_4^{3-}	K^+
1 h	0.5PK	Y=47.42*X-1.137	Y=3.842*X-0.05451	Y=16.02*X-0.3447	0.9727	0.9860	0.9799
	PK	Y=56.87*X-1.806	Y=4.750*X-0.01902	Y=25.23*X-0.7851	0.9839	0.9667	0.9804
	2PK	Y=62.75*X-2.255	Y=5.816*X + 0.01839	Y=35.12*X-1.327	0.9854	0.9877	0.9725
2 h	0.5PK	Y=117.9*X-4.438	Y=12.68*X-0.5279	Y=51.83*X-2.568	0.9156	0.7828	0.9056
	PK	Y=135.7*X-5.001	Y=14.71*X-0.3853	Y=61.52*X-2.162	0.9626	0.9783	0.9892
	2PK	Y=164.4*X-6.852	Y=17.75*X-0.3735	Y=78.59*X-2.758	0.9751	0.9864	0.9893
4 h	0.5PK	Y=261.6*X-10.74	Y=35.31*X-1.692	Y=112.9*X-5.487	0.9536	0.8237	0.9034
	PK	Y=355.8*X-16.59	Y=35.78*X-0.9367	Y=141.9*X-5.712	0.9570	0.9675	0.9865
	2PK	Y=416.1*X-20.17	Y=47.72*X-1.445	Y=167.2*X-5.659	0.9720	0.9678	0.9777

Table S2. The correlations between nutrient absorptions (NH_4^+, PO_4^{3-} and K^+) and root surface area under different concentrations of P and K (0.5PK, PK, and 2PK).

Time	Treatment	Matched curve			R^2		
		NH_4^+	PO_4^{3-}	K^+	NH_4^+	PO_4^{3-}	K^+
1 h	0.5PK	Y=1.783*X - 5.523	Y=0.1424*X - 0.3982	Y=0.6092*X - 1.864	0.7024	0.6916	0.7233
	PK	Y=1.569*X-3.552	Y=0.1342*X-0.1829	Y=0.7178*X-1.682	0.7277	0.7501	0.7708
	2PK	Y=1.905*X-5.295	Y=0.1855*X-0.3190	Y=1.148*X-3.542	0.5940	0.6570	0.6802
2 h	0.5PK	Y=3.351*X-7.282	Y=0.4064*X-1.091	Y=1.554*X-4.270	0.6382	0.6946	0.7026
	PK	Y=4.420*X-10.81	Y=0.4482*X-0.8398	Y=1.938*X-4.426	0.7461	0.6633	0.7174
	2PK	Y=5.492*X-17.69	Y=0.6045*X-1.615	Y=2.627*X-7.949	0.6613	0.6949	0.6719
4 h	0.5PK	Y=7.355*X-16.62	Y=1.118*X-3.181	Y=3.349*X-8.997	0.6509	0.7125	0.6865
	PK	Y=11.80*X-32.98	Y=1.102*X-2.107	Y=4.608*X-11.69	0.7658	0.6680	0.7563
	2PK	Y=14.01*X-48.28	Y=1.615*X-4.717	Y=5.673*X-17.22	0.6695	0.6732	0.6835

Table S3. Primers used for qRT-PCR assay.

Gene Name	Forward (5'-3')	Reverse (5'-3')
OsAMT1;1 (Os04g0509600)	TCTCTTCTACGGGCTCAAGAAGC	CAAATTTATGACGTGACGATCGAGA
OsAMT1;2 (Os02g0620600)	GATCTACGGCGAGTCGGGCACGAT	TTCCATCTCTGTCGAGGTCGAGACG
OsPHT1;1 (Os03g0150600)	CGCTTCCGTACGAGTGGTAGT	GGTTCTTTCAAATCCAGGGAAA
OsPHT1;2 (Os03g0150800)	AGCTGTTGGGTCGCCTTTACTACA	ACGACCATGAGGATGAGCGTGAAT
OsHAK1 (Os04g0401700)	GTTGATGATGCTGATGTTGGAAG	CCAACACTTTCAGCTGAAAC
OsHAK5 (Os01g0930400)	ATTGTGGACTATTTTGAAAGAA	CATTGTGGACTATTTTGAAAGAA
OSA1 (Os03g0689300)	ACTGAGCCAGGCCTT AGTGT	GTATCCACCCAGCACAACTC
OsACTIN	TCAAATGCTAGCTGCACCAC	AGCACGGCTTGAATAGCG
